# Tumor and Peritoneum-Associated Macrophage Gene Signature as a Novel Molecular Biomarker in Gastric Cancer

**DOI:** 10.3390/ijms25074117

**Published:** 2024-04-08

**Authors:** Kevin M. Sullivan, Haiqing Li, Annie Yang, Zhifang Zhang, Ruben R. Munoz, Kelly M. Mahuron, Yate-Ching Yuan, Isaac Benjamin Paz, Daniel Von Hoff, Haiyong Han, Yuman Fong, Yanghee Woo

**Affiliations:** 1 Department of Surgery, City of Hope National Medical Center, Duarte, CA 91010, USA; sullkevi@gmail.com (K.M.S.); ayang@coh.org (A.Y.); zhzhang@coh.org (Z.Z.); kmahuron@coh.org (K.M.M.); bpaz@coh.org (I.B.P.); yfong@coh.org (Y.F.); 2 Integrative Genome Core, Beckman Research Institute, City of Hope National Medical Center, Duarte, CA 91010, USA; hali@coh.org (H.L.); yyuan@coh.org (Y.-C.Y.); 3 Department of Computational and Quantitative Medicine, City of Hope National Medical Center, Duarte, CA 91010, USA; 4 Molecular Medicine Division, Translational Genomics Research Institute, Phoenix, AZ 85004, USA; rmunoz@tgen.org (R.R.M.); dvh@tgen.org (D.V.H.); hhan@tgen.org (H.H.); 5 Cancer Immunotherapeutics Program, Beckman Research Institute, City of Hope National Medical Center, Duarte, CA 91010, USA

**Keywords:** gastric cancer, peritoneal metastases, macrophage, liquid biopsy, biomarker

## Abstract

A spectrum of immune states resulting from tumor resident macrophages and T-lymphocytes in the solid tumor microenvironment correlates with patient outcomes. We hypothesized that in gastric cancer (GC), macrophages in a polarized immunosuppressive transcriptional state would be prognostic of poor survival. We derived transcriptomic signatures for M2 (M2_TS_, *MRC1*; *MS4A4A*; *CD36*; *CCL13*; *CCL18*; *CCL23*; *SLC38A6*; *FGL2*; *FN1*; *MAF*) and M1 (M1_TS_, *CCR7*; *IL2RA*; *CXCL11*; *CCL19*; *CXCL10*; *PLA1A*; *PTX3*) macrophages, and cytolytic T-lymphocytes (CTL_TS_, *GZMA*; *GZMB*; *GZMH*; *GZMM*; *PRF1*). Primary GC in a TCGA stomach cancer dataset was evaluated for signature expressions, and a log-rank test determined overall survival (OS) and the disease-free interval (DFI). In 341 TCGA GC entries, high M2_TS_ expression was associated with histological types and later stages. Low M2_TS_ expression was associated with significantly better 5-year OS and DFI. We validated M2_TS_ in prospectively collected peritoneal fluid of a GC patient cohort (*n* = 28). Single-cell RNA sequencing was used for signature expression in *CD68*^+^*CD163*^+^ cells and the log-rank test compared OS. GC patients with high M2_TS_ in *CD68*^+^*CD163*^+^ cells in their peritoneal fluid had significantly worse OS than those with low expression. Multivariate analyses confirmed M2_TS_ was significantly and independently associated with survival. As an independent predictor of poor survival, M2_TS_ may be prognostic in primary tumors and peritoneal fluid of GC patients.

## 1. Introduction

Gastric cancer (GC) is the fifth most common cause of cancer worldwide, with nearly 1 million new cases per year and over 650,000 deaths per year [1]. GC consists of genomically and immunogenically heterogeneous tumors with a poor 5-year overall survival (OS) of 30% in locally advanced stages and 5% in patients with distant disease [2]. Tumor immunogenicity predicts the response to immunotherapeutic agents in solid tumors, and specifically in gastric adenocarcinoma, an immunosuppressive tumor microenvironment (TME) is associated with poor survival and poor therapeutic response to immune checkpoint inhibitors (ICI) [3]. Combined with traditional chemotherapeutic regimens, ICIs, including pembrolizumab and nivolumab, have improved patient survival and are approved for first-line therapy in unresectable advanced or metastatic GC [4,5,6]. Furthermore, immunotherapy has evolved to include a broader scope of targeted antitumor immune modalities, including chimeric antigen receptor T-cells (CAR T-cells), [7] tumor-infiltrating lymphocytes (TIL), and oncolytic viruses [8]. These strategies highlight the importance of defining immune-associated molecular profiles of cancer for a more accurate assessment of patients’ expected outcomes than that provided by the clinical or pathologic tumor, node, metastasis (TNM) stage alone.

Multi-omic studies have deeply characterized the complex and dynamic TME, revealing the direct effects of immune cell phenotypes on tumor growth and treatment responses. Both immunohistochemical (IHC) and transcriptomic analyses have identified T-cell and macrophage infiltrates in primary GC TME [9,10,11]. IHC staining to identify macrophages of either the inflammatory M1 or immunosuppressive M2 type have shown improved OS in patients whose tumors have a lower CD68^+^ cell population, [12] and CD45^+^CD68^+^ infiltration was higher in GC patients with peritoneal metastases (PM) [13]. Furthermore, tumor growth in a murine xenograft model was greater when GC cells were co-inoculated with M2 macrophages [13]. A meta-analysis of studies from Asia and Europe investigating tumor-associated macrophages (TAMs) in GC showed no correlation between CD68^+^ cell density and patient OS. However, the included studies with various definitions of M2 macrophages showed that a greater infiltration of M2 TAMs was associated with poorer patient OS [14]. A higher IL-6 and elevated CD163^+^ population were associated with worse disease-free survival (DFS), disease-specific survival (DSS) [15], or higher-stage disease [16]. However, the macrophage phenotype does not exist in a dichotomy of distinct M1 or M2 states. Rather, macrophages exist along a dynamic polarizing spectrum that exhibits plasticity from one phenotype to another [17,18]. TAMs also demonstrate mixed phenotypes that are difficult to classify as distinctly M1 or M2 due to exposure to multiple types of stimuli in the TME [19], and single markers such as IHC-based CD163 alone are not reliable M2 macrophage markers [20]. This diversity in TAM and macrophage polarization has necessitated a more accurate description of macrophage capacity using a combination of markers [21].

IHC staining has shown a correlation between certain M2 macrophage protein markers and survival. However, due to the differing phenotypic states of macrophages, it is not clear if the functional state of macrophages on a transcriptional level in the TME is associated with survival. In addition, the correlation of immunosuppressive macrophages in the peritoneum TME with survival is not known. We aimed to determine whether a highly immunosuppressive phenotype of M2 macrophages in primary tumors was associated with survival and to further characterize the role of this macrophage phenotype in the peritoneum. From whole genome expression profiles of macrophages and T-cells of peripheral blood of healthy donors, we selected three gene panels representing proinflammatory M1 macrophages, severely immunosuppressive M2 macrophages, or cytolytic T-cells. We determined the clinical relevance of these transcriptional signatures for each cell type in primary tumors and peritoneal fluid from GC patients.

## 2. Results

### 2.1. Derivation of the Immune Cell Subset-Specific Gene Panels as Transcriptomic Signatures of GC Outcomes

A group of macrophage-associated genes representing a spectrum of polarized M1 and M2 immune states was previously identified in peripheral blood monocytes of healthy donors [22]. We selected genes with the highest and lowest differential expression of M1/M2 ratio of macrophage polarization and defined them as the gene panels for M1-specific or M2-specific transcriptional signatures, respectively. To diversify the function of genes included in the panels, we selected the top 2–3 differentially expressed genes from each category of membrane receptors, cytokines and chemokines, solute carriers, enzymes, extracellular mediators, and DNA-binding factors if the ratio of M1/M2 differential expression (or vice versa) was greater than 10-fold. *CCR7*, *IL2RA*, *CXCL11*, *CCL19*, *CXCL10*, *PLA1A*, and *PTX3* genes were selected to define the M1 transcriptomic signature (M1_TS_), whereas *MRC1*, *MS4A4A*, *CD36*, *CCL13*, *CCL18*, *CCL23*, *SLC38A6*, *FGL2*, *FN1*, and *MAF* genes were used as the M2 transcriptomic signature (M2_TS_). The literature was reviewed for independent studies in which genes identified for each signature were investigated to confirm the functional significance of each gene in macrophages. We confirmed the functional or phenotypic association of each selected gene in the signature in independent studies. In M1_TS_, each of the genes was associated with inflammatory or anti-tumoral functions of macrophages in local TMEs (*CCR7* [23,24], *CXCL11* [23,25], *CCL19* [26,27], *CXCL10* [23,25], *PLA1A* [25], *PTX3* [25,28]). For M2_TS_, additional studies in primary or metastatic tumors similarly were consistent with immunosuppressive or pro-tumoral functions (*MRC1* [23,29,30], *MS4A4A* [31,32], *CD36* [23,33,34], *CCL13* [35], *CCL18* [29,36,37], *CCL23* [38,39], *SLC38A6*, *FGL2* [40,41], *FN1* [42,43], *MAF* [30]). We also used granzyme and perforin-associated genes, *GZMA*, *GZMB*, *GZMH*, *GZMM*, and *PRF1*, to define a gene signature representing a highly active cytolytic T-lymphocyte (CTL_TS_) population involved in T-cell mediated antitumor immunity.

### 2.2. Highly Immunosuppressive M2_TS_ Is Associated with Negative Prognostic Clinical Factors

**Figure 1:**
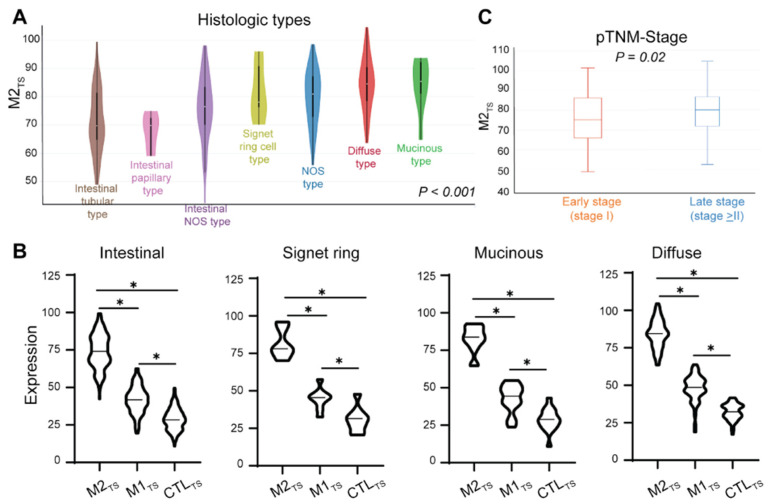
Association of M2-defining macrophage gene expression with histologic subtype and pTMN stage. (**A**) Histologic subtypes for intestinal adenocarcinomas, diffuse adenocarcinoma, mucinous adenocarcinoma, and signet ring cell adenocarcinoma are associated with M2-defining signatures. One-way ANOVA, *p* < 0.001. (**B**) Within each histologic subtype, M2-defining macrophage signature expression is significantly greater than both M1-defining and T-cell cytolytic signature expression. *t*-test for each comparison, * *p* < 0.05. (**C**) Greater M2-defining macrophage expression is associated with more advanced stages. *t*-test, *p* = 0.02. M2_TS_, M2 transcriptomic signature. NOS type, not otherwise specified type. TNM, tumor node metastasis. M1_TS_, M1 transcriptomic signature. CTL_TS_, cytotoxic T-lymphocyte transcriptomic signature.

We analyzed 341 primary GC samples in the TCGA stomach cancer database. We observed that high M2_TS_ expression levels were significantly associated with known negative prognostic factors, such as histological classifications of signet ring cell (SRC), diffuse, and mucinous type gastric adenocarcinoma in the TCGA stomach cancer cohort (*p* < 0.001, Figure 1A). Conversely, intestinal type gastric adenocarcinoma (intestinal tubular, papillary, and not otherwise specified (NOS) type) was associated with lower M2_TS_ expression levels. A similar pattern was seen for M1_TS_, where intestinal type histology appeared to have lower M1_TS_ expression levels than SRC, mucinous, and diffuse types (*p* < 0.001, Supplementary Figure S1A). Higher levels of CTL_TS_ expression were associated with diffuse and SRC gastric adenocarcinoma. In comparison, intestinal and mucinous types had lower CTL_TS_ expression (*p* = 0.01, Supplementary Figure S1A). Regardless of histologic subtype, however, all primary gastric tumors had relatively high M2_TS_ expression compared to M1 and CTL_TS_ (Figure 1B).

To evaluate immune signatures and their association with the patient’s GC stage, we defined early GC (EGC) as pathologic stage I and advanced GC (AGC) as pathologic stage II and III based on the 8th edition GC TNM-staging guidelines. We found that lower M2_TS_ expression levels were significantly associated with EGC, whereas AGC had significantly higher M2_TS_ expression (*p* = 0.02, Figure 1C). Higher M1_TS_ expression was also associated with later stages (*p* = 0.002, Supplementary Figure S1B). No significant difference was observed of CTL_TS_ expression in early versus late stages (*p* = 0.06, Supplementary Figure S1B).

### 2.3. High M2_TS_ Expression in Primary GC Is Associated with Poor Survival

**Figure 2:**
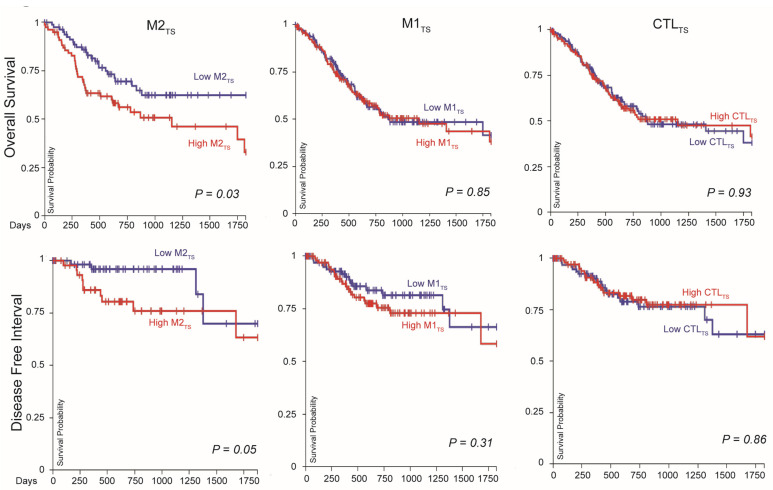
M2-defining macrophage, but not M1-defining or T-cell cytolytic, signature is associated with survival. The highest quintile of M2-defining macrophage signature expression is associated with worse OS (*p* = 0.03) and DFI (*p* = 0.05). High levels of M1-macrophage and T-cell cytolytic signature expression were not associated with OS or DFI (*p* > 0.05). OS, overall survival. DFI, disease free interval. M2_TS_, M2 transcriptomic signature. M1_TS_, M1 transcriptomic signature. CTL_TS_, cytotoxic T-lymphocyte transcriptomic signature.

Because higher M2_TS_ expression was associated with diffuse type and SRC histology and higher pathologic stages, we evaluated whether M2_TS_ expression was related to patient outcomes. We found that the lowest quartile of M2_TS_ expression was significantly associated with better 5-year OS (*p* = 0.03) and DFI (*p* = 0.05) compared to the highest quartile of M2_TS_ expression (Figure 2). We found no significant association of OS or DFI with M1_TS_ expression and CTL_TS_ expression (Figure 2). Additionally, we found no significant association of OS or DFI with either *CD68* or *CD163* expression alone, nor a significant association of OS or DFI with a combined expression of all signatures (Supplementary Figure S2).

### 2.4. Single-Cell RNA Sequencing Demonstrates Different Populations of M2 Macrophage Polarization

**Figure 3:**
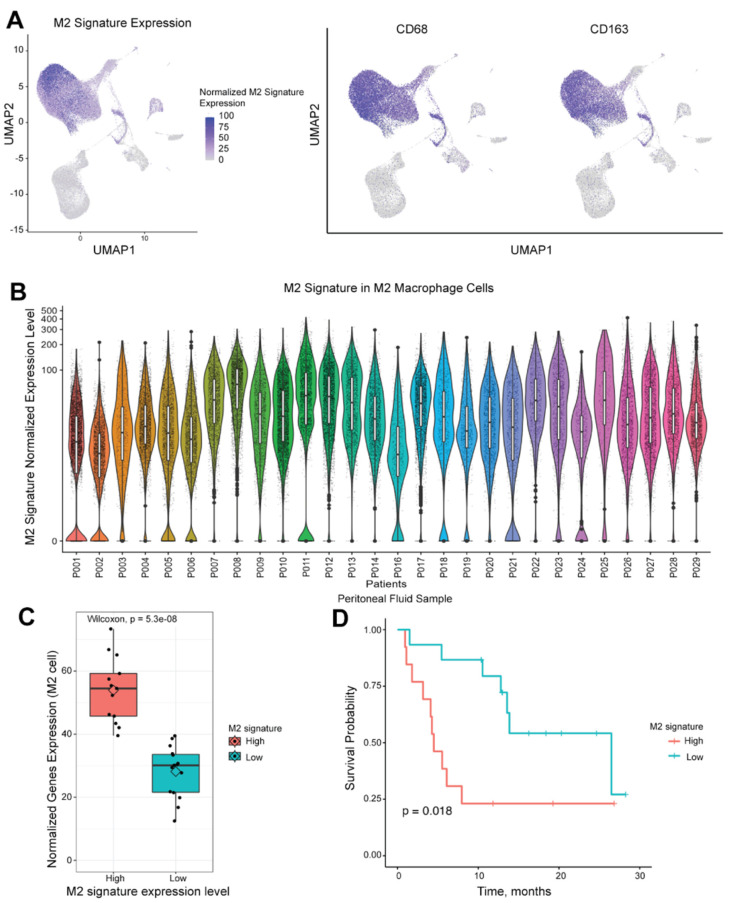
M2-defining signature is expressed in the M2 macrophage cell cluster on single-cell analysis of peritoneal fluid samples and is associated with survival in peritoneal fluid samples. (**A**) UMAP plot displays the cells with higher M2-defining signature expression as purple dot, which is primarily seen in the M2 macrophage cells (*CD68*^+^*CD163*^+^). (**B**) The expression profile of the M2-defining macrophage signature is plotted on the y-axis (log scale) using the single-cell RNA seq expression per a single sample. Each dot represents one single-cell M2-defining signature expression level. Cells tend to cluster in subtypes of M2 macrophages of varying expression of the M2-defining macrophage signature. (**C**) High (*n* = 13) and low (*n* = 15) cohorts can be defined from our entire cohort. (**D**) High expression of M2-defining macrophage gene signature in M2 defined cells within peritoneal samples is associated with worse overall survival (*p* = 0.018). UMAP, unform manifold approximation and projection.

We used scRNA-seq to determine M2_TS_ expression levels of each immune cell in the peritoneal fluid (washings or ascites) from prospectively collected samples. We confirmed that the M2_TS_ signature was primarily expressed in cells defined as M2 macrophages using *CD68* and *CD163* expression (Figure 3A). When plotting M2_TS_ expression in each cell per sample, clusters of the signature gene panel expression levels were seen representing M2 cell subtypes with similar transcription of immunosuppressive genes. The expression level of M2_TS_ within *CD68*^+^*CD163*^+^ cells was found to exist across a continuum within each patient’s peritoneal fluid sample, with several samples containing greater numbers of macrophages with high signature expression (Figure 3B). This finding demonstrates heterogeneity of polarization of cells across samples and the diversity of macrophage polarization states across a spectrum of M2 immunosuppressive transcriptional gene expression. Of the 28 patients, scRNA-seq identified 15 patients with high M2_TS_ expression and 13 with low M2_TS_ expression (Figure 3C).

The GC peritoneal fluid cohort included ethnically diverse patients with heterogeneous primary tumors with and without cytology-positive disease or peritoneal carcinomatosis. Table 1 shows patient demographics, including age, gender, ethnicity, and tumor characteristics, such as histology, molecular features, and TNM stage. No clinical patient- or tumor-specific factors were associated with M2_TS_ expression.

### 2.5. M2_TS_ in Peritoneal Macrophages Is Associated with OS in GC Patients

Finally, we found that M2_TS_ expression levels in the peritoneal fluid were associated with OS of GC patients. Within *CD68*^+^*CD163*^+^ cells, samples with high M2_TS_ expression had poorer OS than patients with low M2_TS_ expression in their peritoneal fluid. Median OS was 4.5 months for M2_TS_ high expressors and 26.5 months for low expressors (Figure 3D, hazard ratio (HR) 3.14, 95% confidence interval (CI) 1.17–8.46, *p* = 0.018). Univariate analysis using both patient and tumor-specific factors to evaluate correlations with survival showed that high M2_TS_ expression was associated with worse survival (Table 2, HR 3.14, 95% CI 1.17–8.46, *p* = 0.024).

Multivariate analysis, including clinical predictors of worse survival in our cohort, showed that high M2_TS_ expression remained independently predictive of worse survival (Table 3, HR 6.58, 95% CI 1.68–25.79, *p* = 0.007) after adjusting for other factors. As expected, stage IV was also predictive of worse survival.

## 3. Discussion

Here, we demonstrate the clinical significance of a distinct immunosuppressive macrophage signature, M2_TS_ (*MRC1*, *MS4A4A*, *CD36*, *CCL13*, *CCL18*, *CCL23*, *SLC38A6*, *FGL2*, *FN1*, *MAF*) in GC patients. Our unique M2_TS_ was derived from the blood of healthy donors and validated as a biomarker in primary and metastatic gastric tumors as a predictor of poor survival. We showed that high M2_TS_ expression in primary GC from the TCGA stomach cancer cohort is associated with negative prognostic clinicopathologic factors in GC. We further validated its prognostic significance as a biomarker predictive of poor survival across stages in a prospectively collected independent cohort of peritoneal fluid from GC patients. These results strongly suggest that M2_TS_-expressing macrophages represent a highly immunosuppressive phenotype associated with more aggressive tumor biology and higher stages (Figure 1). Further, this transcriptional signature was also associated with survival in the primary GC cohort from TCGA. Elevated expression of the immunosuppressive M2-macrophage signature was independently predictive of survival for patients with GC. Previous studies using few surface markers [12,13,14,15] to define macrophages as M1 versus M2 are limited by the potential for a spectrum of functional polarization in immunosuppressive or inflammatory capability. Our findings highlight the biological and functional importance of the combination of genes for membrane receptors (*MRC1*, *MS4A4A*, *CD36*), cytokines and chemokines (*CCL13*, *CCL18*, *CCL23*), solute carriers (*SLC38A6*), extracellular mediators (*FGL2*, *FN1*), and DNA binding factors (*MAF*) that make up the M2_TS_ which are highly differentially expressed in M2-specific macrophages and highlight the importance of macrophage function in both the primary GC and peritoneal microenvironments.

The M2_TS_ assessment in the TCGA stomach cancer cohort provides significant insights into the immune states of the four genomically stable (GS) molecular subtypes of GC [44] that can predict survival and response to therapy [45]. Diffuse type GC is predominately categorized in the GS molecular subtype. GS diffuse type of GC with SRC features has a higher risk of peritoneal recurrence after curative therapy and is relatively resistant to immunotherapy. RNA transcriptional data from the TCGA stomach cancer dataset show that our M2_TS_ is identifiable in 341 entries of primary GC, and high M2_TS_ expression is associated with diffuse type GC. These results add to the current knowledge of the absence of immunogenicity in diffuse phenotypes of GS tumors. M1 and cytolytic T-cell gene signatures were also more likely to be present in diffuse type and SRC GC compared to other histologic types (Supplementary Figure S1). While it is difficult to discern the molecular drivers of poor outcomes by looking at one time point of transcriptomic immune activity, overall, our findings suggest a potentially more immunologically active milieu in diffuse and SRC GC compared to intestinal GC. However, comparing the expressions of each immune subset gene signatures within various primary histologies demonstrates that M2_TS_ is the most highly expressed of the immune signatures (Figure 1B). Moreover, only M2_TS_ was significantly associated with OS, while M1_TS_ and CTL_TS_ were not (Figure 2). Therefore, the immune milieu of GC appears polarized in a M2_TS_-dominant state that drives the overall condition of the TME.

The association of M2_TS_ with diffuse type and the poor prognosis associated with high M2_TS_ expression in peritoneal fluid are consistent with the current understanding of GCPM, which is the leading cause of therapeutic failure and death. Our prospectively collected peritoneal fluid samples included a heterogenous GC patient population who was both naïve to treatment or received multiple lines of systemic therapy and had either the presence or absence of gross peritoneal tumors, malignant ascites, occult peritoneal cytology positive disease, or fluid cytology negative for malignant cells. Using scRNA-seq analyses of immune transcriptomic signatures, we identified patients with high or low expression of M2_TS,_ including multiple subsets of macrophages expressing varying M2_TS_. We then validated high M2_TS_ expression in the peritoneal fluid to be associated with poorer OS than patients with lower expression levels (Figure 3). Through univariate analysis, we verified that high M2_TS_ expression is significantly associated with an increased mortality risk (Table 2). Multivariate analysis, including the stage, history of curative resection, tumor differentiation, and microsatellite status, identified M2_TS_ as an independent factor of worse survival (Table 3). These findings highlight the negative impact of a severely immunosuppressive M2 transcriptional state in the peritoneum with regards to disease progression and patient death.

Studies have shown that TAM infiltration in primary GC, defined by IHC staining of single markers such as CD68, is associated with worse survival, possibly by increasing invasiveness via β-catenin [46]. TAMs, defined as CD163^+^ cells in the tumor stroma and at the margin, were associated with worse survival. These TAMs expressed elevated levels of CXCL12 [47], a chemokine found in immunosuppressive microenvironments [48], suggesting an immunosuppressive function of macrophages. IHC staining has demonstrated an association of TAMs with survival in GC [12,13,14]. However, each of these studies has used 1–2 surface staining markers such as either CD68 or CD163 to define M2 macrophages and therefore do not describe the functional or specific phenotype of TAMs. The use of these markers to define M2 macrophages is nonspecific as there are multiple subtypes of M2 macrophages that are CD68^+^ or CD163^+^ with varying immunosuppressive capacity [49]. Conversely, M2_TS_, a measure of transcriptional expression using genes from multiple functional categories, better describes the functional status of M2 immunosuppressive macrophages. The finding of *CD68*^+^*CD163*^+^ macrophages from our peritoneal sample cohort with a wide range of M2_TS_ expression (Figure 3B) is consistent with a continuum or spectrum of macrophage polarization, with M2_TS_ high cells representing a highly immunosuppressive polarized subset of *CD68*^+^*CD163*^+^ macrophages. In our study, expression of single genes *CD68* or *CD163* was also not associated with survival (Supplementary Figure S2). However, combining markers to define polarization of macrophages is likely an improved way to delineate the most immunosuppressive M2 macrophages [21], and M2_TS_ combination of genes was predictive, while the single TAM genes, likely representing a range of functionally diverse macrophages, were not.

In a previous single-cell transcriptional characterization of GCPM, more M2 macrophages were found in tumors defined as ‘gastric dominant’ versus ‘GI mixed’, and the ‘gastric dominant’ tumors had worse survival [50]. Another multiplex profiling of PM from GC [51] demonstrated a lower relative fraction of monocytes in diffuse GC compared to intestinal and lower monocyte fraction in SRC compared to NOS samples. These findings suggest that monocyte or macrophage functional polarization and phenotype, rather than total population number or fractional proportion, are the primary driver of the immune microenvironment. The study also described ascites samples characterized as a ‘mesenchymal-like’ phenotype with elevated TIM-3 expression and a higher fraction of cytolytic lymphocytes compared to ‘epithelial-like’ phenotypes; the ‘mesenchymal-like’ phenotype had a greater incidence of non-response to chemotherapy. This higher level of TIM-3 checkpoint expression, despite the presence of cytolytic lymphocytes, suggests an immunosuppressive driver in patients with a poor prognosis, given the lack of response to chemotherapy. These studies are, therefore, overall consistent with our findings of the association of immunosuppressive macrophage transcriptional expression with survival, although we have more directly demonstrated this association both in primary GC and peritoneal samples.

One limitation of our study is some incomplete clinical data regarding the PD-L1 and microsatellite status of some patients, as these were not routinely tested on every patient during the collection period. Another limitation is that it is challenging to discern molecular drivers of poor outcomes by looking at a single time point of transcriptomic immune activity. Our results suggest a potentially more immunologically active milieu in diffuse and SRC GC than intestinal GC, given the elevation of each signature level. However, they also indicate that an M2_TS_ dominant state, not consisting of the amount of infiltrating immune cells but rather the transcriptomic status of immune cells, affects OS of GC patients. An additional limitation is the inability to determine whether these tumor and histological classifications directly cause the increased immunosuppressive phenotype of the M2 macrophages or vice versa. But the association of the M2-defining macrophage transcriptional signature with these poor prognostic factors is consistent with its prognostic ability of OS and DFI in primary GC. Another study has shown an association of diffuse histology with an immune desert immunophenotype, lacking significant T-cell infiltration within the tumor [10], which would allow for increased immunosuppressive influence from macrophages in tumors with diffuse histology.

Identification of this transcriptional signature provides opportunities to improve prognostic accuracy and guide the design of future interventional trials. Several drugs are currently under preclinical development and early-phase clinical trials that aim to repolarize M2 macrophages to immune-activating M1 macrophages. For example, colony-stimulating factor 1 (CSF1) or CSF1 receptor (CSF1R) blockade has been shown to repolarize M2 to M1 macrophages and increase tumor sensitivity to other immunotherapies [52,53]. Clinically available drugs targeting CSF1 or CSF1R include pexidartinib, which repolarizes M2 macrophages [54] and has demonstrated safety and activity in a phase 3 trial [55] resulting in the first available systemic therapy available for tenosynovial giant cell tumors. Another type of CSF1 therapy, cabiralizumab, has shown safety in combination with other immunotherapies in a phase 1 trial for several cancer types [56]. A different strategy of macrophage polarization targets the C-C motif chemokine ligand 2 (CCL2)/C-C motif chemokine receptor 2 (CCR2) axis with available drugs such as carlumab, which was well tolerated but not effective in a phase 2 trial of efficacy in metastatic castration-resistant prostate cancer [57]. Another novel strategy uses anti-CD47 antibodies that block the “do not eat me” CD47 and signal regulatory protein alpha (SIRPα) interactions that allow for tumor cell evasion of phagocytosis. Clinically available anti-CD47 therapeutic antibodies include Hu5F9-G4, which was safe and tolerated in phase 1 trials in lymphoma [58] and solid tumors [59]. Several clinically available drugs targeting metabolic pathways used in other diseases, including perhexiline and VLX600, have been shown to repolarize M2 to M1 macrophages [60]. Preclinical studies of alternative therapies such as BMS-794833 [61] or mannose receptor (CD206) conformational switching by RP-182 [62] have shown the ability to repolarize macrophages. They can be studied to determine the effects on macrophages and, ultimately, GC patient outcomes.

The M2_TS_ signature can potentially be used as a prognostic biomarker using samples collected during either the biopsy of the primary tumor during endoscopy, or during diagnostic laparoscopy already routinely performed for staging newly diagnosed GC [42], obviating the need for a new procedure or specimen collection. As our prospective cohort included cytology-negative patients, elevation of the M2_TS_ macrophage transcriptional signature in the peritoneal fluid could potentially represent an even more sensitive prognostic indicator of GC outcomes than cytology; future work will compare cytology status with M2_TS_ macrophage signature in prognostic ability. Nonetheless, the addition of M2_TS_ macrophage transcriptional status to other prognostic factors, including stage, histology, and cytology status, may allow for a more accurate overall prediction of disease biology and influence the aggressiveness and modality (for example, chemotherapy versus immunotherapy) of treatment. Future work will evaluate changes and responses in M2_TS_ status after treatment with chemotherapy, immunotherapy, or both. Because the transcriptional signature includes many genes involved in immunosuppressive functions of macrophages, one or a combination of the associated M2_TS_ genes may be suitable for future investigations targeting this subset of macrophages.

In conclusion, our study highlights the clinical significance of a novel M2_TS_ in GC associated with a severely immunosuppressive immune phenotype in the local, regional, and systemic TME. The collection of genes representing immunosuppressive functions of macrophages directly implicates a specific phenotype of macrophages in poor prognosis, compared to previous studies that may include a spectrum of macrophage functions. We propose M2_TS_ as a promising and versatile immune biomarker predictive of GC patient outcomes across TNM stages that can be obtained from specimens already collected during diagnosis and staging of GC. High M2_TS_ expression can be evaluated by next-generation sequencing of biopsy specimens obtained from the primary tumor or transcriptomic evaluation of “liquid biopsies” of blood and peritoneal fluid and enrich novel therapeutic interventions for M2 targets.

## 4. Materials and Methods

### 4.1. The Cancer Genome Atlas (TCGA) Stomach Cancer Dataset Analysis

Gene expression profiles for primary gastric adenocarcinomas were obtained from the TCGA stomach cohort and analyzed using the University of California Santa Cruz (UCSC) Xena visualization web tool (https://xena.ucsc.edu, accessed on 15 August 2021). [63]. We applied filters to the dataset to exclude low sample size (defined as less than five entries), “discrepancy”, or “unknown entries” before analysis.

### 4.2. Prospective Cohort of Peritoneal Samples 

Ascites or peritoneal washing samples were prospectively collected from 28 patients undergoing diagnostic laparoscopy or paracentesis (diagnostic or therapeutic) for biopsy-proven gastric adenocarcinoma (stage I–IV) with or without peritoneal metastases. Inclusion criteria were an age greater than 18 years old and known diagnosis of gastric cancer. The exclusion criterion was the inability to give consent. Gross peritoneal disease was neither considered inclusion nor exclusion criteria. During diagnostic laparoscopy, peritoneal specimens were collected after lavage with ~1000 mL of normal saline with laparoscopic access. For GC patients with peritoneal carcinomatosis and malignant ascites, up to 5 L of the discarded ascites collected during a paracentesis was obtained. The first 80 mL–160 mL of the specimens was sent to pathology for routine cytologic evaluation. The remaining samples were immediately placed on wet ice for single-cell processing. The fluid was spun down, and the cell pellet was collected and resuspended in phosphate-buffered saline (PBS, pH 7.4). The cell count was obtained and prepared for single-cell RNA sequencing (scRNA-seq) using the Chromium Single-Cell 3′ Gene Expression System from 10X Genomics (Pleasanton, CA, USA).

### 4.3. Single-Cell RNA Sequencing

Single cell whole transcriptome profiling of peritoneal cells was performed using the Chromium Single-Cell 3′ Gene Expression system from 10X Genomics. Single cells were resuspended in PBS buffer at 10^6^ cells/mL and loaded onto Chromium chips. Single-cell capturing, barcoding, and cDNA library preparation were performed using the Chromium Single-Cell 3′ Library & Gel Bead Kit v2 (10X Genomics, Pleasanton, CA, USA) per manufacturer protocol. The final sequencing libraries were checked for quality on Agilent 4200 Tapestation System (Santa Clara, CA, USA) and quantified by fluorometry staining (QuBit) assay. Libraries were sequenced on a HiSeq4000 (Illumina, San Diego, CA, USA) at a depth of ~50,000 reads per cell. The scRNA-seq was analyzed using standard Seurat v3 [64] integration workflow. Top 2000 variated features were selected to find anchors between pairs of datasets. The integrated dataset was dimension-reduced and visualized using the Uniform Manifold Approximation and Projection (UMAP) with top 30 principal components. All cells are clustered using the original Louvain algorithm. Dot plots, violin plots, and heatmaps were generated using the R Seurat package (version 5). The scRNA-seq data are available at Gene Expression Omnibus (GEO), GSE228598.

### 4.4. Single-Cell Type Identification

We performed a two-layer analysis to identify single cells from the peritoneal biospecimen that were macrophages. First, we used well-established macrophage markers (*CD68*^+^), then performed gene clustering analysis to determine differential gene clusters within the macrophages that differentiate M1 (*CCL19*^+^*CCR7*^+^), M2 (*CD68*^+^*CD163*^+^), and others that did not fall into the M1 and M2 clusters. Finally, we investigated the survival differences between patients whose peritoneal cells overexpressed M2_TS_ and those who did not.

### 4.5. Statistical Analysis

Histological subtypes and signature expressions were evaluated using ANOVA or *t*-test where appropriate. We defined *p* < 0.05 as significant. Each transcriptional signature was quantified as a composite total expression of total gene expression within the signature. Peritoneal samples were stratified into high (≥39.23 counts per 10K (CP10K)) or low (less than 39.23 CP10K) M2_TS_ expression of these selected genes based on total expression of transcriptional signatures. The cutoff point (39.23 CP10K) was determined by the lowest *p*-value in the log-ranking test using CutoffFinder (http://molpath.charite.de/cutoff, accessed on 10 July 2022) [65], a systematic optimization method for biomarker cutoff determination. Comparisons of clinicopathologic characteristics between patient groups (M2_TS_ High vs. M2_TS_ Low) were analyzed using Fisher’s exact test for categorical variables, and Wilcoxon test for continuous variables. For both TCGA and the scRNA-seq analysis, we used a log-rank test to compare Kaplan–Meier curves for OS and the disease-free interval (DFI), comparing the highest and lowest quartiles of transcriptional signature expression in the TCGA analysis. Univariate Cox regression analysis was performed to determine significant factors for survival. Based on the univariate analysis result, we used the significant factor M2_TS_ signature and microsatellite status for the multivariate Cox regression analysis model. The factors of resection status, stage, and differentiation which were close to significant (*p* ≤ 0.1) or significant (*p* < 0.05) on univariate were included in the multivariate model.

## 5. Conclusions

Macrophages and T-lymphocytes each perform important interactions and functions in the GC TME. This study has demonstrated that a highly immunosuppressive phenotype of macrophages, defined by high expression of the combination of genes, predicts poor outcomes when present in both primary GC and the peritoneum of GC patients. While understanding the proportions of each cell type is important, a deeper understanding of the functional phenotype of the cell populations present is critical to be able to potentially design therapies for the most influential cell types or predict the biology of the disease or response to therapy.

## Figures and Tables

**Table 1:** Clinicopathologic characteristics by M2_TS_ expression levels.

		M2_TS_ Expression		*p*-Value
		Low *n* = 15 (%)	High *n* = 13 (%)	
Age (years)	≤40	6 (40.0)	1 (7.7)	0.086
	>40 and ≤65	4 (26.7)	8 (61.5)	
	>65	5 (33.3)	4 (30.8)	
Age (years)	Mean ± SD	51.7 ± 20.2	60.7 ± 13.6	0.184
Gender	Female	9 (60.0)	4 (33.3)	0.322
	Male	6 (40.0)	8 (66.7)	
Ethnicity	Asian	5 (33.3)	4 (30.8)	0.288
	Black	1 (6.7)	0 (0.0)	
	Hispanic	6 (40.0)	3 (23.1)	
	Non-Hispanic White	2 (13.3)	6 (46.2)	
	Other	1 (6.7)	0 (0.0)	
Cytology	Negative	6 (40.0)	4 (30.8)	0.91
	Positive	9 (60.0)	9 (69.2)	
Lauren Classification	Diffuse	11 (73.3)	10 (76.9)	1.0
	Intestinal	4 (26.7)	3 (23.1)	
Differentiation	Moderately	0 (0.0)	2 (15.4)	0.144
	Poorly	15 (100.0)	10 (76.9)	
	Well to moderately	0 (0.0)	1 (7.7)	
Signet Ring Cell	Absent	5 (33.3)	8 (61.5)	0.266
	Present	10 (66.7)	5 (38.5)	
Microsatellite Status	MSI high	1 (6.7)	0 (0.0)	0.55
	MSS	12 (80.0)	12 (92.3)	
	Unknown	2 (13.3)	1 (7.7)	
PD-L1	Negative	4 (26.7)	5 (38.5)	0.52
	Positive	10 (66.7)	6 (46.2)	
	Unknown	1 (6.7)	2 (15.4)	
Stage	I–III	2 (13.3)	2 (15.4)	1.0
	IV	13 (86.7)	11 (84.6)	
Serum Albumin	<3 g/dL	4 (26.7)	5 (38.5)	0.794
	≥3 g/dL	11 (73.3)	8 (61.5)	

M2_TS_, M2 transcriptomic signature. SD, standard deviation. MSI, microsatellite instable. MSS, microsatellite stable.

**Table 2:** Univariate Cox regression analysis of survival in the peritoneal fluid cohort.

		Patients (%)	HR (95% CI, *p*-Value)
M2_TS_ Expression	Low	15 (53.6)	ref
	High	13 (46.4)	3.14 (1.17–8.46, 0.02)
Age (years)	≤40	7 (25.0)	ref
	>40 and ≤65	12 (42.9)	3.67 (0.79–17.05, 0.097)
	>65	9 (32.1)	2.51 (0.50–12.53, 0.26)
Age (years)	Mean ± SD	55.9 ± 17.7	1.01 (0.99–1.04, 0.33)
Gender	Female	13 (48.1)	ref
	Male	14 (51.9)	0.96 (0.36–2.57, 0.93)
Ethnicity	Asian	9 (32.1)	ref
	Black	1 (3.6)	3.21 (0.35–29.23, 0.30)
	Hispanic	9 (32.1)	0.93 (0.25–3.49, 0.92)
	Non-Hispanic White	8 (28.6)	1.76 (0.53–5.79, 0.35)
	Other	1 (3.6)	0.00 (0.00–Inf, 1.0)
Resection	No	17 (60.7)	ref
	Yes	11 (39.3)	2.31 (0.89–6.02, 0.09)
Stage	I-III	4 (14.3)	ref
	IV	24 (85.7)	5.53 (0.69–44.07, 0.106)
Lauren Classification	Diffuse	21 (75.0)	ref
	Intestinal	7 (25.0)	1.53 (0.56–4.18, 0.40)
Differentiation	Moderately	2 (7.1)	ref
	Poorly	25 (89.3)	0.23 (0.05–1.09, 0.06)
	Well to moderately	1 (3.6)	NA
Microsatellite Status	MSS	24 (85.7)	ref
	MSI high	1 (3.6)	15.04 (1.34–169.06, 0.028)
	Unknown	3 (10.7)	2.36 (0.66–8.50, 0.19)

HR, hazard ratio. CI, confidence interval. M2_TS_, M2 transcriptional signature. Ref, reference value. SD, standard deviation. MSS, microsatellite stable. MSI, microsatellite instable. Bolded *p*-value indicates statistical significance (*p* < 0.05).

**Table 3:** Multivariate Cox regression analysis of survival from the peritoneal fluid cohort.

		Patients (%)	HR (95% CI, *p*-Value)
M2_TS_ Expression	Low	15 (53.6)	ref
	High	13 (46.4)	6.58 (1.68–25.79, 0.007)
Resection	No	17 (60.7)	ref
	Yes	11 (39.3)	2.30 (0.67–7.98, 0.19)
Stage	I-III	4 (14.3)	ref
	IV	24 (85.7)	14.71 (1.11–194.42, 0.041)
Differentiation	Moderately	2 (7.1)	ref
	Poorly	25 (89.3)	0.98 (0.16–6.11, 0.98)
	Well to moderately	1 (3.6)	4 × 10^9^ (0.00–Inf, 1.000)
Microsatellite Status	MSS	24 (85.7)	ref
	MSI high	1 (3.6)	145.33 (6.55–3225.37, 0.002)
	Unknown	3 (10.7)	3.67 (0.74–18.18, *i*)

HR, hazard ratio. CI, confidence interval. M2_TS_, M2 transcriptional signature. Ref, reference value. MSS, microsatellite stable. MSI, microsatellite instable. Bolded *p*-value indicates statistical significance (*p* < 0.05).

## Data Availability

The data accessed and utilized from primary gastric cancer samples is available from The Cancer Genome Atlas Project. The single-cell RNA sequencing data supporting the conclusion of this article are available in the Gene Expression Omnibus Repository, GSE228598.

## Supplementary Materials

The following supporting information can be downloaded at: https://www.mdpi.com/article/10.3390/ijms25074117/s1.
