# Supplementary material for: Tumor and Peritoneum-Associated Macrophage Gene Signature as a Novel Molecular Biomarker in Gastric Cancer
Source: Int J Mol Sci. 2024 Apr 8;25(7):4117. doi: 10.3390/ijms25074117 (PMC11012629; doi:10.3390/ijms25074117)
Supplement: Supplementary file 1 [file ijms-25-04117-s001.zip › ijms-2902402-supplementary.pdf]

SUPPLEMENTAL FIGURES:

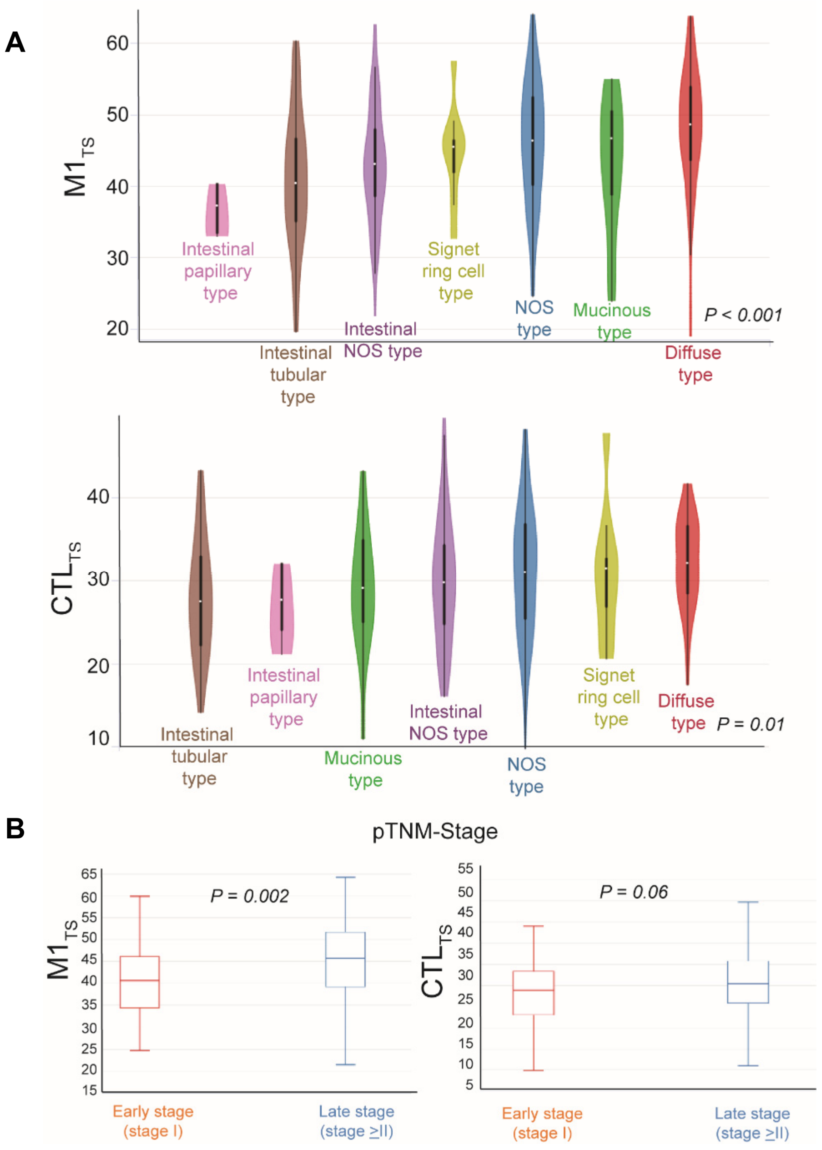

Supplemental Figure S1. (A) Histologic subtype for intestinal adenocarcinomas, diffuse adenocarcinoma, mucinous adenocarcinoma, and signet ring cell adenocarcinoma is associated with M1-defining signature and T cell cytolytic signature. (B) Greater M1-defining macrophage expression, but not T cell cytolytic signature expression, is associated with higher stage.

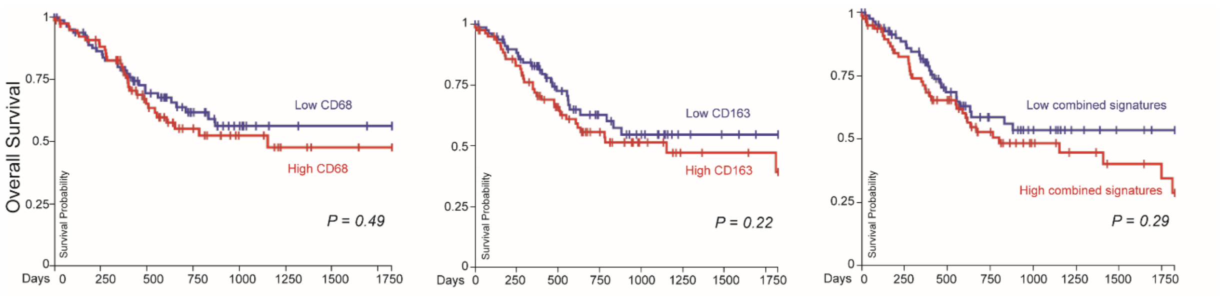

Supplemental Figure S2. In primary gastric cancer samples from the TCGA, there is no significant difference in overall survival for single tumor-associated macrophage genes *CD68* and *CD163* nor for combined expression of all $M1_{TS}$, $M2_{TS}$, and CTL_{TS}.
